# The Push and Pull of Land Use Policy: Reconstructing 150 Years of Development and Conservation Land Acquisition

**DOI:** 10.1371/journal.pone.0103489

**Published:** 2014-07-30

**Authors:** Maria João Santos, Terry Watt, Stephanie Pincetl

**Affiliations:** 1 Center for Spatial and Textual Analysis, Spatial History Project, and Bill Lane Center for the American West, Stanford University, Stanford, California, United States of America; 2 Terry Watt Planning Associates, San Francisco, California, United States of America; 3 California Center for Sustainable Communities, Institute of the Environment University of California Los Angeles, Los Angeles, California, United States of America; University of KwaZulu-Natal, South Africa

## Abstract

The growth of human populations and their resource needs have stressed the conservation of natural land resources. Many policies and programs have been implemented to address the pressures on land resources and notwithstanding this pressure, significant acquisition of land for conservation has occurred throughout history in the U.S., and internationally. Here we assess the on-the-ground result of the evolution of land use policies in California as a pioneer forerunner, in the form of acquisition of land for conservation (i.e. Open Space), and its impact on the rest of the U.S. and beyond. To this end we describe the timeline and spatial representation of the growth of California’s conservation network over the last 150 years, and link it to the history of land use policies. We then assess whether conservation land acquisition has consistently grown through time or occurred in specific decades. About ¼ of the state is now designated Open Space. Fewer and larger areas conserved and acquired at the beginning of the 20^th^ century; the conservation network was complemented with a larger number of smaller sized properties. Despite acquisition of land in every decade, the process was uneven (E = 0.3 for California, E = 0.14±0.08 average for the state’s counties), mostly due to the large acquisitions and land set asides in the 1900s, followed by 1930s and 1940s. This process was a result of a comprehensive set of legislation that evolved through time, and resulted from the competing needs for development and conservation. Even with the impressive 174,000 km^2^ of public lands in California, the future of California’s natural infrastructure and natural heritage cannot rely solely on these public lands, nor public agencies and their resources. Critically a future course of land preservation relying on the purchase of new lands – in California and beyond – for conservation is tremendously expensive.

## Introduction

The growth of human populations and their resource needs have stressed the conservation of natural land resources. Conservation planning has focused on moving beyond opportunism [1], and is using strategic planning to identify the best spatial solutions to meet conservation goals, and implement such solutions on-the-ground [2]. This is evident in the recent approaches to conservation of the Nature Conservancy, for example, identifying the most valuable and endangered conservation hotspots across the globe, and investing funds in those places to preserve land. However, temporal aspects have been somewhat neglected (but see [3]), only currently coming to play as changes in land cover/land use and climate may threaten the persistence of the already existing conservation networks [4]. This, of course raises difficult scientific and ethical issues beyond the scope of this paper, about moving fauna and flora to locations where they will survive under climate change conditions and other types of interventions. At the same time, the creation of a conservation network can in itself induces change [5], and this change can be tied with historical legacies or periods of intense action [3]. These periods can be identified in the timeline of protected area establishment and in the creation of key conservation legislation, but integration of both timelines is yet to be conducted. While the former is easily assessed worldwide, the latter is affected by local, regional, national and international contexts. Here we tie both timelines, to understand the push-and-pull of land use policies over the last 150 years, using California as case study to demonstrate the importance of the integration of both timelines and to suggest such research be conducted in other places. We focus on California for its preeminence in this area, and its international significance. California is arguably the state that led the implementation of the concept of resource reserves since the 19^th^ century, in the country and elsewhere [3].

Forty four percent of the Earth’s plant species and 35% of the Earth’s terrestrial vertebrates are endemic to 25 hotspots, despite the fact these hotspots make up just 1.4% of the world’s land cover [6]. These biodiversity hotspots have immense implications for conservation strategies to protect large number of species living in relatively small regions of land. They also represent lands that have been and are highly threatened by land cover and climate changes. The California Floristic Province is one of the world’s 25 most biodiverse areas on the planet [6]. For California itself, 44% of its plant and vertebrate species are endemic to the state [7]. Today, ¼ of California are properties designated as Open Space. Open Space refers to “*lands protected through title ownership by a public agency or non-profit land conservation organization”* [8]. Open Spaces includes a wide array of types of properties from City Parks to National Parks, and some heavily managed land, such as that managed by the federal Bureau of Land Management (BLM). Fee title ownership by the public exists for all of these Open Spaces. However, Open Space does not include private land in easement contracts. Moreover, lands in public ownership – aside from the National Park lands – are subject to the Federal Land Policy and Management Act, FLPMA (1976) that allows multiple-use of public lands and for the most part does not recognize conservation as an exclusive use except on lands with designations such as wilderness.

Already in the later decades of the nineteenth century, preservation of California’s remarkable natural resources was underway. In 1864 Yosemite Valley and the Mariposa Grove of Giant Sequoia trees were protected by Abraham Lincoln, with a grant of 80.9km^2^ of federal land. At the time, the federal government maintained ownership of vast lands, part of the public domain, that had not been sold through the Homestead Act (1865) and other acts that distributed the lands the federal government inherited with its new territories. Much of California’s early preserved land was land that belonged to the federal government and was “withdrawn” from sale to the public, and instead, conserved as part of the public domain. This eventually became the backbone of the lands of the National Park Service, and the National Forest Service. Similar patterns of land reservation occurred in many states of the American West. Nevada, for example, is 80% public lands, or federal ownership. In southern California, the San Gabriel and San Bernardino Forest reserves were created in 1892 and 1893 respectively, from the public domain by President Harrison, some of the first land reservations from sale from the public lands in the nation. California is in many ways the state that led the implementation of the concept of resource reserves since the 19^th^ century [9], lobbying Congress and the President about the public interest in the public lands, and that they should be conserved. State interest in land conservation was reflected in 1927 by the then recently created State Parks Commission that instructed Frederick Law Olmsted Jr. “*to make a survey to determine what lands are suitable and desirable for the ultimate development of a comprehensive, well-balanced state park system, and to define the relation of such a system to other means of conserving and utilizing the scenic and recreational resources of the state*”[10]. The survey was conducted, and set the vision, for example, for the creation of the state’s Redwood Parks. Birth home of the Sierra Club, there is no question that interest in protecting the state’s scenic beauty has been important for nearly a century and a half, an idea exported to other parts of the country and the world to establish their own conservation timelines [3], [9].

In this paper we review the history of land conservation in California from a land use and governance perspective, and assess the on-the-ground result of the evolution of land use policies in the state in the form of acquisition of land for conservation. To do so we reconstruct the conservation history of California, that is, the growth of the conservation network per decade over the last 150 years, and link it to the timeline of land use policies as an example for further research of this type. We assess whether conservation land acquisition has taken place relatively evenly over time or has been concentrated in specific decades. We then review the challenges of current land use regulations, including fiscal constraints and regulatory requirements, which create a complex terrain for further land conservation. New tools are emerging for protection but structural constraints that we will discuss, remain in place as well, and they will differ from state to state and from nation to nation. In this discussion we develop a method that can be replicated to better help understanding both the ways in which lands have been conserved and the potential pathways for further conservation given the structural conditions that exist in California.

## Materials and Methods

### Open Space land

To obtain geospatial data for all Open Spaces in the state, we used the California Protected Area Database (CPAD) as a base database [8]. CPAD was developed by GreenInfo Network as a GIS inventory of all fee-protected Open Space properties in California. We selected this database and not the World Database on Protected Areas [3], [11] because CPAD has a more comprehensive representation of the Open Space properties in the state, from City Parks to National Parks. It does not include all public land, nor private owners, or properties protected through the use of easements. Neither WDPA nor CPAD included information on the acquisition and establishment dates of each and every Open Space property in California or other protected lands across the globe [8], [11]. To complement this information gap, and reconstruct a conservation history for the state, we contacted federal and state agencies and Non-Governmental Organizations that manage Open Space properties to request information. We asked for acquisition date, that is, when the fee title was purchased, and establishment date – when the property was open to the public. Below we present statistics for data acquisition for the great majority of the properties and area of the state designated as Open Space. In the remaining analysis we use date of acquisition for our timeline of conservation land acquisition. To understand the conditions under which land were protected, knowing the timelines of establishment is important, as it allows better insights into the reasons why such acquisitions might have occurred [3].

### Land use policies

To complement, compare and contrast the Open space acquisition timeline we developed the land use history timeline. We reviewed the legal documents that detailed land use policies in California. These included primary source legislation and published literature on examples, applications and assessments of the impacts of such legislation (for example see http://ceres.ca.gov/planning/state.html). Key literature was searched for the date of the legislation, its description, intended targets, and when possible implications of the legislation for land use planning and Open Space acquisition.

### Data analysis

The timeline of conservation land acquisition describes the cumulative area acquired per decade in the state. To assess whether each decade contribution to the conservation network was higher or lower than the state’s average, we calculated the slope of the regression line of all the decades and compared it with the slope of the regression for consecutive decades. Significantly different decades were illustrated in the state’s timeline. We then tested whether the acquisition of land was even throughout time for the state and how it compared across counties. We calculated the Simpson evenness index (E) which varies from 0 (uneven distribution of conservation action through the decades) to 1 (equal distribution of conservation action through the decades; [12]). We calculated evenness for the state and for each county, and determined whether counties differed from the state values. We then linked the timeline of conservation land acquisition with the history of land use policy in the state. Because of the link between funding for land acquisition and development, we analyzed in detail the most populous counties in the beginning of the 20^th^ century.

## Results

California has 16,000 Open Space properties in the form of 53,337 parcels that were acquired throughout the last 150 years. These properties cover ¼ of the state’s area (112,156km^2^). We were able to retrieve information on acquisition date for a total of 35,807 properties (67%), corresponding to 110,300km^2^ (98.5%) of the state’s Open Space. Merced, Orange, San Joaquin, Stanislaus, and Sutter were the counties from which it was harder to collect information. City Parks were the properties that had the least data about date acquired and/or created.

### History of conservation land acquisition

**Figure 1:**
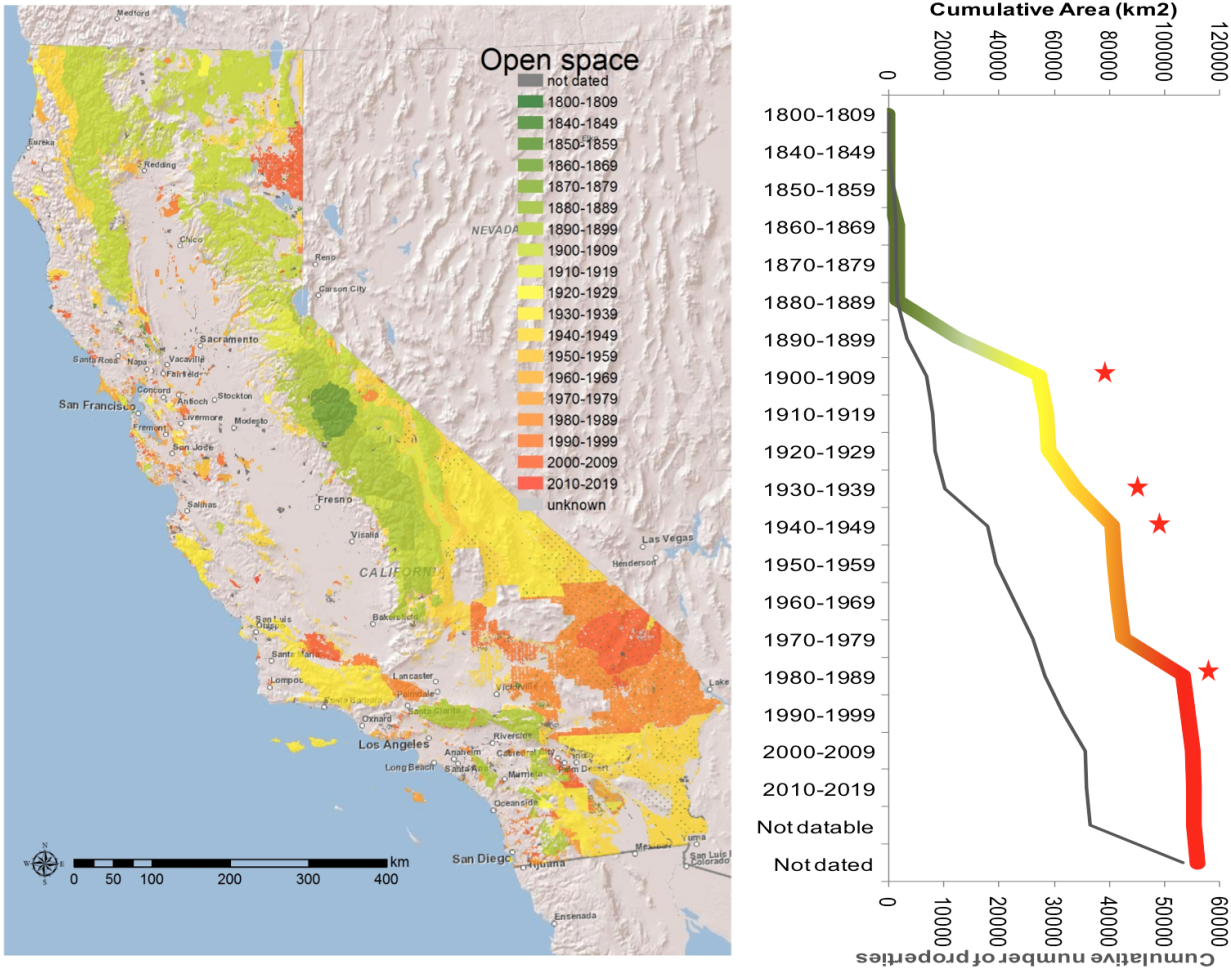
Timeline of Open Space acquisition and establishment in California over the last 150 years. Left panel – spatial representation, Right panel – timeline (red stars represent the decades where the conservation land acquisition was higher than the average over all the decades).

Large tracts of land were set aside from the remaining public domain at the end of the 19^th^ century. These were the mountain forest lands to be managed by the subsequently created United States Forest Service (Figure 1). Land acquisitions in the 1880s and 1890s contributed with an area of 30,000km^2^, 30% of the current Open Space land. With the close of the public domain and into the twentieth century, additional land had to then be acquired for parks (mostly by federal agencies) as it was privately owned. Geographically, earlier land preservation focused on mountain regions, mostly in the Central and Southern Sierras, and the mountains surrounding Los Angeles (Figure 1). Land set asides and early acquisitions were predominantly in the northern California mountain ranges until 1920s (Figure 1). In the 1930’s and 1940’s, large tracts in southern California were purchased, as for example the lands for Death Valley and Joshua Tree National Parks. At this time the California State Parks further contributed to land acquisition, a pattern that continued into the 1950’s (Figure 1). These acquisitions were also greatly aided by regulations passed in the 1940’s that allowed Special Districts (a type of government unit that is formed to provide a specific service, such as schools, sewers, street lighting, parks or other) to acquire conservation lands. From 1950 to 1980 more scattered and smaller sized properties filled in the conservation network, as the larger tracts of land were already public. However, this pattern shifted from the 1980’s to today with the acquisition of large tracts of desert lands (Figure 1).

**Figure 2:**
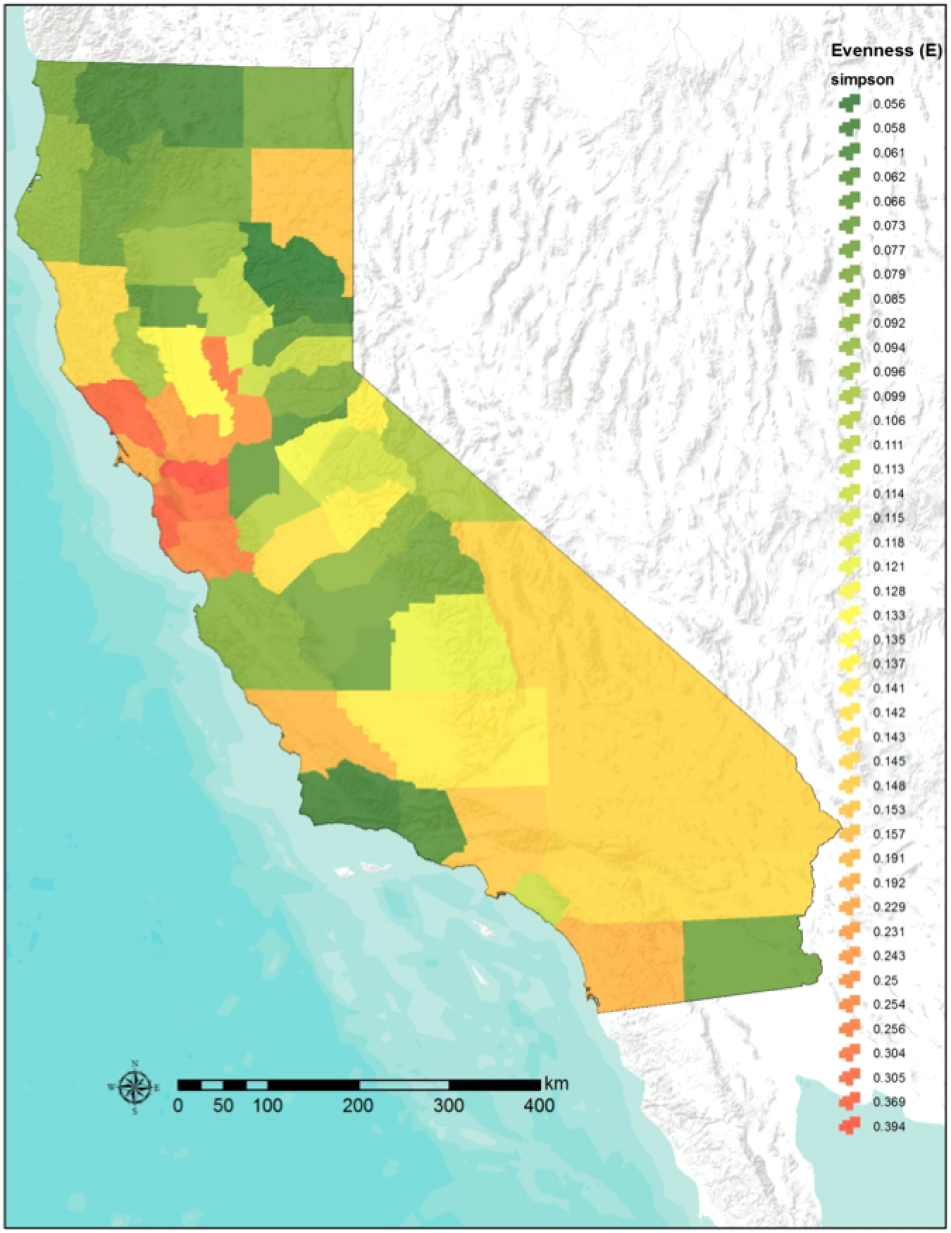
Per county evenness of the conservation timeline. Lighter colors represent higher evenness. The higher evenness is achieved around the San Francisco Bay area, and most of the counties show an evenness half of that of the state (E_California_ = 0.3).

The rate of land acquisition (slope of the regression line for the state conservation acquisition) was 6874. 8 km^2^ per decade (Figure 1). This rate was significantly higher in five decades (1890–1900, 1900–1910, 1920–1930, 1930–1940 and 1970–1980, indicated in Figure 1 with red stars), with acquisition rates ranging from 9697km^2^ in 1930–1930, to 29799km^2^ in 1900–1910, a very steep acquisition slope 5 times higher than the state’s rate (Figure 1). The rate of conservation land acquisition in California over the last 150 years was also spatially uneven. The state’s evenness value was 0.3, and 0.15 as the counties average (Figure 2). Plumas, Siskiyou and Santa Barbara counties were very uneven in their conservation land acquisition (Figure 2), while the Bay Area counties had a more even acquisition rate over time, and greater or equal to that of state. This is because of the greater amount of Open Space area acquired or set aside in the 1900’s, followed by the 1930’s and 1940’s, with rates up to 5-times greater than the state’s average rate of land acquisition (red stars in Figure 1).

**Figure 3:**
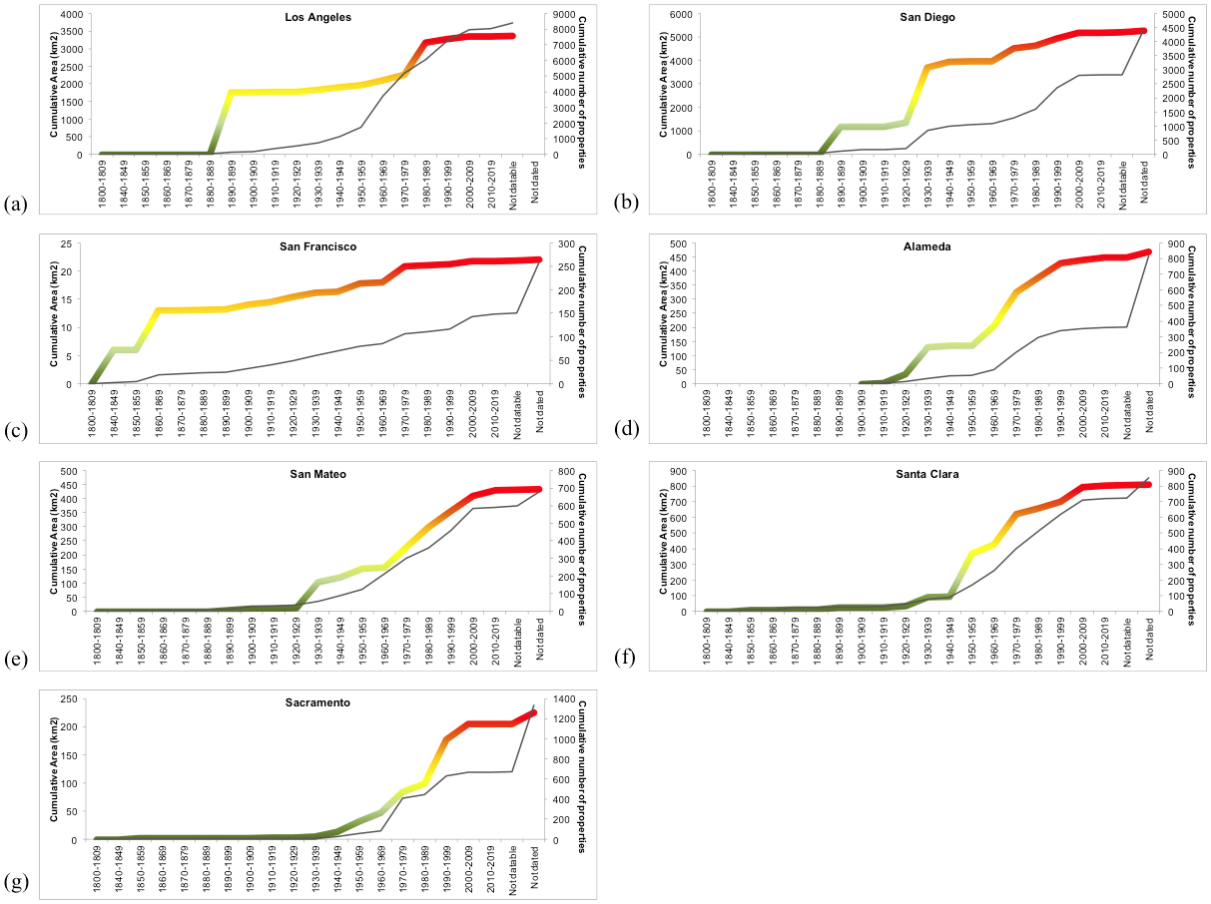
Timeline of conservation for the most populous counties in California in 1910.

Of the most populous counties in 1910, San Francisco engaged in some of the earliest acquisitions and then continued a constant rate of acquisition throughout the twentieth century (Figure 3). Los Angeles conservation land grew more slowly at the beginning of the twentieth century, showed a stepwise pattern. High federal involvement at the turn of the twentieth century and again in 1970–1990, was supplemented by land acquisitions by nonprofit organizations. San Diego shows a big increase in land acquisition in 1920’s followed by a smooth rate thereafter; this is a similar pattern to Alameda, San Mateo and Santa Clara, but the peak of Open Space acquisition in these counties starts in 1950’s. The most populous counties show, however, a very different proportion of conservation land; the largest being in San Diego and the smallest in San Francisco and Sacramento (Table 1).

**Table 1:** Proportion of county land in Open Space.

County	County area (km^2^)	Open Space area (km^2^)	Area of county in Open Space (%)
Los Angeles	10242.41	3375.847	32.96
San Diego	10973.57	5286.147	48.17
San Francisco	277.02	22.12	7.98
Alameda	2126.87	469.39	22.07
San Mateo	1430.55	435.12	30.42
Santa Clara	3378.21	811.63	24.03
Sacramento	2580.21	225.94	8.76

Selected counties represent the most populous counties as in 1910s.

### History of land use policies

Local control over land use was instituted very early on to ensure autonomy of localities from state interference. This local independence was staunchly solidified by 1914 state legislation authorizing charter cities to make and enforce all laws and regulations in respect to municipal affairs ([13]; Table 2). This includes by 1937, the requirement to adopt comprehensive plans, which are guiding documents that set out a general vision for how the land in the jurisdiction of the city or unincorporated in the county should be utilized. Extensive land development patterns were facilitated by urban and state policies including uniform subdivision regulations in 1929 [14], [15], [16], [17], [18], and attempts to establish and enforce General Plans to guide development were overwhelmed by the tenacity of local developers and the proliferation of new municipalities, each with its own planning authority [14]. Most of the growth between 1950 and 1960 was located in suburban communities [19], aided by the recently built highways. By the early 1960s, state policy makers and Governor Pat Brown were concerned about growing fragmentation at the local level, including the increasing numbers of special districts formed to service urban growth. The state legislature created a state Planning Office in 1959. Governor Pat Brown appointed the Coordinating Council on Urban Policy to come up with policy proposals to improve planning and coordination of growth. The Coordinating Council’s report urged the creation of one multipurpose district in each of the state’s metropolitan areas to deal with regional issues such as air pollution, water supply, sewage, parks and more. These proposals were roundly resisted by the Chamber of Commerce, cities and counties. In 1963, the state legislature, instead, created watered down county-level Local Agency Formation Commissions who were empowered to approve or disapprove any petition for incorporation, special-district formation or dissolution, and annexation in each county.

**Table 2:** The push-and-pull of development and Open space land use policies in the state.

*The push and pull of development and natural resources legislation*		
*Natural resources*		*For development*
	**1914 – Charter cities**	Authorizes the making and enforcement of all laws and regulations for municipal affairs
	**1937 – Comprehensive (General) Plans**	Sets out a general vision for the land in the jurisdiction of the city or county, and how it should be utilized
	**1947 – State Highway Act**	Authorize and promote the development of highways
	**1956 and 1962 – Federal Highway Act**	Authorize and promote the development of highways
Sets out a general vision for the water in the jurisdiction(s) and how it should be utilized	**1957 – California Water Plan**	
	**1962 – California Tomorrow**	Regional government to manage growth in the state
Stop the infill of the San Francisco Bay	**1965 – Bay Area Conservation and Development Commission**	
Allows contracts between the state and farmers to preserve agriculture lands	**1965 – Williamson Act**	
Developers to set aside a portion of their subdivisions as Parks or Open Space, or pay fees for parkland acquisition	**1965 – Quimby Act**	
	**1970 - Open Space in General Plans**	Requires that General Plans include Open Space elements
Protocol of analysis and public disclosure of environmental impacts of proposed projects, and measures for mitigation	**1970 – California Environmental Quality Act**	
	**1971 – General Plans**	Become required, not voluntary
For water protection	**1972 – California Wild and Scenic Rivers Act**	
For coastal area protection	**1972 – Coastal Protection Act**	
For protection of endangered species and their habitat	**1972 – Endangered Species Act**	
	**1978 – Proposition 13**	Property tax reduction initiative
	**1978 – AB 857**	Curb urban sprawl and directing new urban growth to existing cities and suburbs – infill
For protection of endangered species habitat	**1982 – Habitat Conservation Plans**	
For protection of natural communities (endangered or not)	**1991 – Natural Communities Conservation Planning**	
Infrastructure Planning	**2001 – AB857 Farmland Protection Act**	
Plan and manage natural communities for global climate change	**2006 – AB32 The Global Warming Solutions Act**	
Greenhouse Gas emissions regulation	**2007 – SB97 CEQA and Greenhouse Gas Emissions**	
	**2008 – SB The Sustainable Communities and climate Protection Act**	Adaptation and vulnerability for human populations

Frustrated by the lack of leadership at the state level, movements to attempt to curb growth and to protect the state’s natural resources mobilized (Table 2). The attempt by the state Highway Commission to improve Highway 101 by cutting through portions of the Prairie Creek Redwoods and Jedediah Smith Redwoods State Parks, was defeated by the Sierra Club that then became a watch dog over projects proposed by the Highway Commission that often targeted land in state ownership, thus a less costly strategy that the purchasing of privately held lands. Another important example was the creation of California Tomorrow in 1962, a nonprofit educational institution that developed influential proposals for reforming state government and for the creation of regional government to manage growth in the state. These environmental concerns contributed to the passage of the 1965 California Land Conservation Act (Williamson Act). The Williamson Act allowed farmers and ranchers to qualify for lower property tax rates if they entered into contracts keeping their lands in agriculture for a minimum of ten years. The 1965 Quimby Act was also passed. It allowed local governments to require developers to set aside a portion of their subdivisions as parks or Open Space, or pay fees for parkland acquisition and maintenance. The 1969 Santa Barbara oil spill had a jarring effect on the state, prompting a number of legislative initiatives, including the passage of the California Environmental Quality Act (CEQA) in 1970. Modeled on the federal National Environmental Policy Act (NEPA), it requires state and local agencies within California to follow a protocol of analysis and public disclosure of environmental impacts of proposed projects, and to adopt feasible measures for mitigating those impacts. It is a mandatory part of every California state and local agency process.

In 1972 state voters created the Coastal Commission, an entity appointed by the Governor, in charge of protecting California’s coast from over development. This was one of the strongest measures to emerge from the first slow growth era [20], and a clear reaction to the increased pace of urbanization that was occurring. Other counteracting effect was that developments were being held up by the federal Endangered Species Act (ESA), passed in 1972 with the aim to preserve charismatic species. Environmental organizations used the ESA to challenge development with some success and developers found themselves stymied and slowed down. ESA challenges were based on a species by species endangerment threat, and it became clear that policies based on protecting individual species would scarcely achieve the goal of preserving species as they relied on habitats that needed to be preserved as well. Habitat conservation plans (HCP’s) were added to the ESA by Congress in 1982 in an effort to address the need to conserve the habitat for imperiled species. Congress viewed HCPs as a win-win situation for imperiled species; HCPs took habitat into consideration, allowing a more encompassing approach, and they created Incidental Take Permits (ITPs), which allowed – for the first time – the taking of a limited amount of habitat in exchange for a commitment to an HCP to protect and manage other habitat areas, ensuring the species’ overall recovery chances [20]. But HCPs still were created to protect one species at time. In response, California, under Governor Wilson, created Multiple Species Habitat Conservation Planning (MSHCP) and the 1991 Natural Communities Conservation Planning (NCCP) in an effort to encompass entire ecosystems and their processes. The NCCP process was intended to bring all stakeholders to the table in order to set aside coherent, regional habitat preserves [18], [21]. NCCPs were endorsed by Secretary of the Interior, Bruce Babbitt as a legitimate implementation of the ESA that would allow the protection of endangered species, but not halt development. This is perhaps the impetus behind the increase in conservation land acquisition in San Diego (Figure 3), one of the precursor counties to develop a NCCP.

All in all, the 1970s were a period of active initiatives to preserve land at the local level with concurrent initiatives aimed to restrict or slow growth throughout the state, but in 1978 state voters passed Proposition 13, the property tax reduction initiative. Local government revenues plummeted from about $10 billion just prior to the passage of Proposition 13 to approximately half of that shortly thereafter. Growth management efforts were hampered by the need for revenues and most counties and cities adopted land use policies most likely to replenish their depleted budgets [14], [18], [22], [23], [24]. The impacts of Proposition 13 likely explain the asymptote of land acquisition rate from 1980s onwards (Figure 1). Some exceptions, however, emerge for counties like Sacramento, where the cumulative rate of conservation land acquisition still increased after 1980s (Figure 3). This is perhaps due to the emergence of creative solutions for raising funds for the acquisition of conservation land. Yet, in addition to Proposition 13 and Proposition 4, voters also passed Propositions 62 and 218 in 1996 extending the supermajority approval requirement of Proposition 13 to virtually all types of assessments, fees or taxes used by local government. This further constricted local government’s flexibility to raise funds for necessary services, let alone to purchase additional lands for conservation purposes (Table 2).

With the decline in local government’s ability to raise money for land preservation through tax dollars, new mechanisms began to be invented. One such new strategy was developed successfully by the Planning and Conservation League (PCL), a statewide environmental organization, which developed a set of inventive campaign funding tactics and ballot initiatives to finance the acquisition and development of park and recreation areas [25]. Nongovernmental organizations and quasi-governmental agencies emerged from this period as new stewards of public spaces, including habitat. State conservancies with budgets funded by grants, donations and foundation funding were created. Recent innovations include the Regional Advanced Mitigation Plan (RAMP), which is an effort to develop a more comprehensive and strategic approach to mitigating biological resource impacts caused by major infrastructure projects, before projects are constructed. RAMPs typically build on a conservation plan that validates and matches the mitigation actions and cumulative impacts. Another emerging concept is the inclusion of Regional Greenprints as an integral part of local General Plans and Sustainable Community Strategies. Greenprints would offer a new way to improve conservation planning by providing a process to map a region’s important Open Space for a full range of ecosystem services including habitat, farmland, recreation, water resources and more. Conservation planning could provide a baseline on which land use plans are developed, and could be linked to CO2 sequestration, a new and popular idea. For example, Preservation Ranch, a 7890ha property in Sonoma County, was just recently purchased with carbon credits funds (http://baynature.org/articles/preservation-ranch-big-conservation-thanks-to-carbon-credits/).

With the advent of climate change, priorities have expanded to include renewable energy and land use that reduces production of greenhouse gas emissions. All of these concerns are embodied in adopted state law and policy. Examples include the 1978 Urban Strategy, AB 857 (Wiggins codified at Section 65041.1 of the Government Code), the 2006 Assembly Bill 32 (California Global Warming Solutions Act), and the 2008 SB 97 (CEQA and Greenhouse Gas Emissions), and Senate Bill 375 (Sustainable Communities and Climate Protection Act).

## Discussion

It can be argued that over the past 150 years a great deal of California’s most scenic landscapes has been preserved. The fundamental dilemma of conserving land is the necessity for land to be purchased. Under the Fifth Amendment of the U.S. Constitution, government must purchase land at its market value, it cannot take land away from land owners, nor force them to sell below the land’s market value. Thus for additional conservation to occur, as the state’s population and cities grow into previously undeveloped areas that have important ecosystems, this land must be purchased from its owners. Moreover, since land development provides localities their revenues, there is a push to continue to zone land for urbanization. Such dynamics are typical throughout the U.S. but in addition, in California, for new developments, fees are assessed on units to create funds to acquire the conservation lands, near or adjacent to the new development – mitigation land. This leads to a paradox – for there to be funds to acquire conservation lands, there needs to be development [26]. In turn this quest for growth limits the land available for conservation – one of the original goals for which growth was planned for.

California’s voluntary approach to meet conservation needs has been a reactive, and accretionary approach that creates obvious mixed messages between local power and regional planning efforts, and mismatches between policy goals and instruments for their implementation. Mixed messages are due to the autonomy of land use designations of cities while conservation needs and habitats do not observe political boundaries [27]. Land use planning is by definition based on the parcelizing of land for development, which is done by cities. This parcelizing then creates sections of land that can be bought and sold individually, allows the land market to work at the municipal and county scale, but this approach fragments the landscape and its governance. While there have been numerous attempts to create regional governments with regulatory authority, and to encourage greater collaboration among local governments [28]; they still have narrow single issue mandates (for example, regional “special districts” for air quality management and flood control). In contrast, conservation needs and habitats do not observe political boundaries, they are regional and even statewide. Preservation of ecosystem requires a landscape or regional approach, keeping parcels together to provide connectivity and or enough space for ecosystems to function well, and to preserve watersheds. Further, conservation lands require revenue to purchase them. Funding streams are often predicated on growth with development fees and various impact fees to finance conservation acquisitions [26]. Thus the state has a paradox: to conserve, there must be development.

There has been a century-long series of efforts to create effective institutions to help guide metropolitan growth and development in California [14], [19], [27]. While early conservation land acquisition tended to occur in the mountain regions, and away from the rapidly growing cities; the push-and-pull of land use policies lead to striking differences in the timeline of the 1910s most populous counties. The early most populous counties took different interpretations of the regulations, and the growth of the conservation network took different shapes. The most steady growth rate is found for San Francisco. San Francisco, along with Alameda, San Mateo, Santa Clara and 5 other counties form the Bay Area, the region that showed the higher evenness in conservation land acquisition. This has been a key region in the state and even an example of conservation success in a growing metropolitan area [28], [29]. In 1965 the Bay Area Conservation and Development Commission was created, a single purpose regional planning agency, to supervise development around San Francisco Bay, and to stop Bay infill projects. The southern California counties show a stepwise growth of the conservation network, associated with the protection of the mountain areas around Los Angeles and desert areas around San Diego.

There are also mismatches between policy goals and instruments for their implementation. California has long recognized the costs of sprawl, and prioritized specific land use outcomes including orderly and efficient land use, infill, farmland protection and conservation of resource lands, but has developed weak planning tools and rules to strongly protect habitat. With the advent of climate change, priorities have expanded to include renewable energy and land use that reduces production of greenhouse gas emissions. These measures aim to curb urban sprawl and directing new urban growth to existing cities and suburbs – infill, revitalizing central cities and neighborhoods, and protecting resource lands, in alignment with the proposals put forward by California Tomorrow and others. While the most recent SB 375 statute is possibly the closest California has come to regional planning and encourages urban and suburban infill, clustered development, mixed land uses, transit oriented development to reduce GHGs [30], it does not change land use law and local control over local land use.

At the same time, other activities are occurring that will also challenge conservation. Paradoxically, concern about GHG emissions is fueling the Governor Jerry Brown’s drive to have a high speed rail line built to link Southern California to the Bay Area and Sacramento. Its route, as currently designed, goes through some of the state’s prime agricultural land, and is argued that it will support even more sub-urbanization in the already urbanizing Central Valley. Governor Jerry Brown is also pursuing more energy resources, including natural gas fracking. A great deal of the state’s natural gas and petroleum resources are also found the Central Valley and will also compete for water, adding another demand in addition to water for agriculture, urban areas and for places of origin. Protected federal lands are not exempt either in California with large scale electricity generation projects in the high desert of the Mohave having been given the green light by the Department of the Interior, despite documented impacts on the desert tortoise among other species.

California has been fortunate to have had magnificent habitats preserved starting in the late 19^th^ century. This is quite different from what has happened in the country and in the world [3], [31]. At the same time, California has had tremendous population growth and urbanization as well. This has led to the push-and-pull in land use policies, creating conflicts between habitat preservation and development. Urban growth is still seen as the key to the prosperity of localities, so there is little incentive to build denser more contained cities and to collaborate across jurisdictions over infrastructure and/or land use. The state legislature, and voters through the ballot initiative process, have passed a number of laws, and created new agencies, to attempt to improve conservation planning and to protect conservation lands. But with the reduction of funding since the early 1980s, conserving lands has been more difficult. Any land purchased by government must be bought at market value, and since land in California is expensive, it means conserving more lands is costly. Innovative approaches to gather conservation acquisition funds are now being proposed and implemented.

Lost perhaps is the bigger vision of how we live on the land over time. Planning law, open space protection processes, finding funding for conservation through development and CO2 markets, are instrumental approaches to an issue that involves much a longer term vision about how people live on the land. Protected areas are not one more land use type – they are a result of a (land use) policy. While protected areas have distinct geographic boundaries they are part of a conservation network, which grows through a dynamic process [32], [33]. U.S. land conservation policies, enabled by the vast area in public ownership, was seen as a model to emulate in many parts of the globe. For example, the International Union for the Conservation of Nature has been at the forefront of land conservation for habitat protection in Africa and increasingly in other parts of the world. Initially this was to simply preserve wildlife, now the mission includes attempting to anticipate the conservation needs due to climate change impacts. In Europe too there are now National Parks, based on the U.S. template, but often include working landscapes [11]. The reconstruction of the history of conservation can then describe the process of growth of a conservation network. This process is yet not well documented [3]; however, the creation of such baseline knowledge can help strategize the maintenance and future additions to the conservation network [33]. Further it can allow us to understand the feedback between land use policies and conservation action on meeting and maintaining conservation goals in California and elsewhere in the world [5], [30], [34].

Perhaps the most important value of historic analyses is what they indicate about the future. Policies could redirect future growth and conservation. As we observed, multiple funding sources contributed to the highest rates of land acquisition in different times in history (federal in 1890–1910, state in 1920–1940, and non-governmental organizations in 1970–1980). We also observed that reduction of development fee funding lead to two outcomes: creativity on methods to raise funds, and strategic acquisitions, perhaps because of the concurrent emergence of systematic conservation planning [1], [2]. Perhaps the reduction of the funding stream imposed by Proposition 13 sparked innovative funding strategies, arguably more sustainable and less paradoxical. As future housing growth depends heavily on economic conditions [35], so do development fees. Innovative funding approaches that do not depend on development fees may be more sustainable in the long run. Linking histories of policies and conservation land acquisition allows reflections on the results of such policies but also on their perpetuity. Over the last 150 years we have seen a push-and-pull between development and conservation policies, some policies remained and some got replaced. The same applies to conservation lands, especially those that are not conserved in perpetuity. The future of these lands holds new challenges, with the onset of climate induced changes. Dynamic reserve systems are being proposed [4], which may take advantage of demoting some of these currently non-in-perpetuity conserved lands, or others, and it also presents challenges on funding sources. Perhaps a RAMP-like strategy can be applied to meet the conservation network dynamics needed to meet climate change-suited conservation. While solutions for the future of conservation are contextual and geographically restricted, the issues of fund raising, land acquisition, development versus conservation, and persistence of the conservation network operate at regional, national and global scales. Only through merging information at such scales can guarantee that the benefits of conservation can be enjoyed by the future generations.
